# I Can’t Sleep! Death Distress, Self-Control, Bedtime Procrastination, and Substance Misuse

**DOI:** 10.1007/s11126-025-10175-1

**Published:** 2025-06-28

**Authors:** Fatma Betul Yilmaz, Seydi Ahmet Satici, Emine Gocet Tekin

**Affiliations:** 1 Department of Psychology, Faculty of Humanities and Social Sciences, Istinye University, Istanbul, Türkiye; 2 Department of Psychological Counselling, Yildiz Technical University, Istanbul, Türkiye; 3 Department of Psychology, Sakarya University, Serdivan, Türkiye

**Keywords:** Substance misuse, Death distress, Self-control, Bedtime procrastination

## Abstract

Substance misuse stands as a critical challenge globally, leading to severe repercussions for individuals and society alike, and placing considerable pressure on public healthcare systems and economic stability. This research delves into the predictive relationships between death distress, self-control, bedtime procrastination, and substance misuse. Engaging a diverse cohort of 479 individuals, including those who consume cigarettes and alcohol, aged between 18 and 71 years (Mean = 34.21, SD = 12.61), the study utilized assessments to gauge death distress, self-control, bedtime procrastination, and substance misuse via self-report questionnaires. Through the application of structural equation modeling, the findings revealed that death distress, self-control, and bedtime procrastination are significant predictors of substance misuse behaviors. Furthermore, the analysis highlights that self-control and bedtime procrastination serve as mediating factors in the relationship between death distress and substance misuse. These findings underscore the necessity of identifying and addressing death distress, self-control, and bedtime procrastination to mitigate the effects of substance misuse, thereby emphasizing their significance in prevention and intervention strategies.

## Introduction

Researchers who study human behavior have long been fascinated by the complicated relationships between psychological elements and how our choices and coping mechanisms work. Among these variables, death distress, self-control, bedtime procrastination, and substance misuse are significant components that interact in complex ways to influence people’s reactions to existential fears and emotional regulation. Death distress, conceptualized as the apprehension and existential concern surrounding mortality, has attracted substantial attention in psychological research [23]. Its profound impact on human behavior extends beyond the realms of philosophical inquiry to intricately influence emotional responses and coping strategies [17]. Notably, research has shown a strong link between death distress and maladaptive behaviors, with substance misuse emerging as a potential coping mechanism [11].

Substance misuse refers to the harmful or inappropriate use of psychoactive substances, including alcohol, illicit drugs, or prescription medications. It encompasses a spectrum of behaviors, from occasional excessive use to addiction, resulting in negative consequences for physical health, mental well-being, and social functioning [6, 22]. The relationship between substance misuse and death distress is complex and encompasses psychological, emotional, and behavioral dimensions. The use of substances as a coping mechanism for existential distress underscores the intricate dynamics between these two constructs. The relationship between substance misuse and death distress highlights the need for comprehensive interventions addressing both psychological well-being and substance-related issues. The literature suggests a complex interplay in which heightened death distress may lead people to turn to substances as a coping mechanism to relieve their distress or temporarily escape existential worries [11]. Conversely, substance misuse, while providing perceived relief from existential worries, often exacerbates anxiety in the long term and perpetuates a cycle of distress [10, 11].

Expanding upon this foundation, the construct of self-control emerges as a crucial element in understanding our proposed sequential chain. The literature underscores the multifaceted role of self-regulation in guiding behavior and managing affective responses [4, 9, 18]. For instance, across nine studies involving 979 participants, findings highlighted the importance of self-regulation in managing fears of mortality. The study results emphasize the vital role of self-control in coping with distressing thoughts and emotions associated with mortality by showing that individuals with higher self-control experienced fewer death-related thoughts, reported lower anxiety, and exhibited reduced sensitivity to mortality cues [3]. Studies like Muraven and Baumeister [12] have explored how anxiety and stress can deplete self-control resources. While not directly related to death distress, this research sheds light on how emotional states might impair self-regulation, potentially supporting the notion that death distress could similarly impact self-control.

Bedtime procrastination, characterized by voluntary delays in sleep initiation despite awareness of negative consequences, assumes significance in this framework [8, 15]. Studies substantiate the association between diminished self-control and procrastinatory sleep behaviors [7, 20], thereby establishing a potential linkage between impaired regulatory capacities and postponement of bedtime. For example, Ahmad et al. [1] found that higher self-control linked to improved sleep quality and reduced bedtime procrastination, while increased bedtime procrastination was associated with lower sleep quality and poorer self-control. Research by Kroese et al. [7, 8] showed that individuals with lower self-control inclinations exhibit a greater tendency to postpone their bedtime routine, even when aware of the negative outcomes associated with insufficient sleep. This insight suggests that individuals struggling with self-control might be more susceptible to engaging in bedtime procrastination behaviors.

The primary objective of this study is to investigate the serial mediation model, examining how self-control and bedtime procrastination collectively mediate the relationship between death distress and substance misuse. In other words, this study aims to explore how death distress, characterized by apprehension or fear related to mortality, might impact an individual’s self-control abilities. Specifically, it seeks to understand whether heightened death distress may be associated with lower levels of self-control, potentially influencing one’s ability to regulate impulses and adhere to bedtime routines which may ultimately result in substance misuse.

## Methods

### Participants and Procedure

In the present study, the participant pool comprised a total of 479 individuals, of which 309 (64.5%) were female, and 170 (35.5%) were male. Regarding marital status, 248 participants (51.8%) were single, whereas 231 (48.2%) were married. In terms of employment, 296 individuals (61.8%) were employed, and 183 (38.2%) were not engaged in any form of employment. The socio-economic status of the participants was categorized into three levels: low, medium, and high. A total of 69 participants (14.5%) were classified under the low socio-economic status, 330 individuals (68.9%) were considered to have a medium socio-economic status, and the remaining 80 participants (16.7%) were classified under the high socio-economic status. Family composition of the participants varied, with 36 individuals (7.5%) being an only child, 167 (34.9%) having one sibling, 161 (33.6%) having two siblings, and 115 (24.0%) reporting to have three or more siblings. Regarding the initiation of substance use, the age of first smoking attempt ranged from 5 to 32 years, with a mean of 17.92 years and a standard deviation (SD) of 3.26. Similarly, the age at which participants first consumed alcohol varied between 5 and 40 years, with a mean age of 18.39 years and a standard deviation (SD) of 3.33 (Table 1).

**Table 1:** Participant information

Variable	Frequency	%
*Gender*		
Female	309	64.5
Male	170	35.5
*Marital status*		
Single	248	51.8
Married	231	48.2
*Employment*		
Employed	296	61.8
Not employed	183	38.2
*Perceived Socio-Economic Status*		
Low	69	14.5
Medium	330	68.9
High	80	16.7
*Number of siblings*		
Only child	36	7.5
Having one sibling	167	34.9
Having two siblings	161	33.6
Having three or more siblings	115	24.0

The survey was distributed by an online platform called Google Forms, and data was collected with convenience sampling. Participants were asked to fill out questionnaires, which are the *Death Distress Scale*,* Brief Control Scale*, Bedtime *Procrastination Scale*,* Profile Index Risk Screening (APIRS)-alcohol*,* and Addiction Profile Index Risk Screening (APIRS)-drug.* Informed consent was obtained before the data collection, and participants were informed that they had the right to withdraw at any time during the survey. Participation was fully voluntary, and participants were not paid. The ethical committee of Yıldız Technical University approved the study (REF: 20231002335).

### Measures

Death distress was measured using the *Death Distress Scale*, which was created by Dadfar and Lester [2] and adopted in Turkish by Yıldırım and Güler [24]. It has nine items (I feel sad when I dream of death.) in three factors, which are anxiety, depression, and obsessive thoughts. The scale is a 5-item Likert scale from 1 (never) to 5 (always). The sum of the items on the same factor gives the total point of that factor. Higher points indicate higher death distress. Internal consistencies were .55 for the anxiety subscale, .68 for the depression subscale, and .88 for obsessive thoughts subscales in the original study [2], and they were as follows: .77, .88, and .91 in the Turkish adaptation study [24].

Self-control was measured using the *Brief Control Scale*, which was created by Tangney et al. [20] and adopted in Turkish by Nebioglu et al. [13]. It has 13 items (I do certain things that are bad for me if they are fun). The scale has one factor in the original study and two factors in Turkish adaptation, which are impulsiveness and self-discipline. The scale is a 5-item Likert scale from 1 (completely disagree) to 5 (completely agree). The sum of the items on the same factor gives the total point of that factor. Internal consistencies were .87 for the impulsivity subscale, .81 for the self-discipline subscale, and .83 for the total scale in the Turkish adaptation [13]. The original study found the internal consistency of the scale to be .89.

Bedtime procrastination was measured via the Bedtime *Procrastination Scale* [7], which was adopted into Turkish by Yılmaz Dinç et al. [25]. The scale is in a one-factor structure with nine items (do not go to bed on time.), which are rated on a scale from 1 (almost never) to 5 (almost always). The internal consistency values are .92 for the original scale and .71 for the Turkish adaptation scale.

Substance misuse was measured on two different scales to differentiate between the use of drugs and alcohol. The scales were P*rofile Index Risk Screening (APIRS)-alcohol* and *Addiction Profile Index Risk Screening (APIRS)-drug which were both developed by* Ogel et al. [14] to measure individuals’ risky substance misuse habits by culturally appropriate tools. *Profile Index Risk Screening (APIRS)-alcohol* has six items (How often have you used alcohol in the last six months?) that are rated in a range from 0 to 1. The scale has two factors. Higher points mean higher alcohol misuse, and 3 or higher points indicate risky alcohol use. The Cronbach alpha value of the scale is .70. *Addiction Profile Index Risk Screening (APIRS)-drug* has 6 items (How often have you used substance in the year?) that are rated on a range from 0 to 1. The scale has a one-factor structure. Higher points mean higher alcohol misuse, and 4 or higher points indicate risky alcohol use. The Cronbach alpha value of the scale is .88.

### Data Analysis

Initially, a correlational study was undertaken to investigate the relationships between substance misuse, death distress, self-control, and bedtime procrastination. Descriptive statistical measures, such as means and standard deviations, were analyzed through IBM SPSS Statistics version 20. The potential mediating effect of self-control and bedtime procrastination was explored through a two-step approach in structural equation modeling. In the preliminary phase, the measurement model was developed to verify the accurate representation of latent constructs by their respective indicators. Following the validation of the measurement model, the structural model was evaluated using maximum likelihood estimation via AMOS Graphics. The evaluation of model fit was based on several indices, including Standardized Root Mean Square Residual (SRMR), Root Mean Square Error of Approximation (RMSEA), Comparative Fit Index (CFI), Normed Fit Index (NFI), Goodness-of-Fit Index (GFI), Tucker-Lewis Index (TLI). According to established guidelines by Hoyle and Panter (1995), a model is regarded as adequately fitting if SRMR and RMSEA are below 0.08, and CFI, NFI and GFI exceed 0.90. Because Bedtime Procrastination Scale is unidimensional with nine items, parceling method was used to decrease measurement errors. Additionally, the significance of mediation models was tested via 5000 bootstrap replicates for direct and indirect effects.

## Results

### Correlations among the Variables

Correlation analysis revealed significant relationships among the variables (see Table 2). Alcohol misuse and drug misuse were positively associated with death distress (*r* =.260 and *r* =.354, respectively, both *p* <.001). Conversely, self-control showed a negative correlation with both alcohol misuse (*r* = −.323, *p* <.001) and drug misuse (*r* = −.285, *p* <.001), Bedtime procrastination was positively correlated with both alcohol (*r* =.354, *p* <.001) and drug misuse (*r* =.328, *p* <.001), as well as with death distress (*r* =.388, *p* <.001). Lastly, there was a significant negative correlation between self-control and bedtime procrastination (*r* = −.409, *p* <.001).

**Table 2:** Descriptive statistics

Variables	Descriptive Statistics and Reliabilities					Correlations			
	Mean	SD	α	ω	λ6	1	2	3	
1. Alcohol misuse	1.115	2.121	0.859	0.868	0.852	–			
2. Drug misuse	0.361	1.284	0.879	0.886	0.854	0.733^**^	–		
3. Death distress	21.505	6.204	0.772	0.760	0.862	0.260^**^	0.354^**^	–	
4. Self-control	32.921	6.025	0.698	0.703	0.704	− 0.323^**^	− 0.285^**^	− 0.315^**^	–
5. Bedtime procrastination	25.144	7.370	0.896	0.899	0.899	0.354^**^	0.328^**^	0.388^**^	− 0.409^**^

Note. ^**^p <.001

### Structural Equation Modelling

The structural equation modeling for our study incorporated four latent constructs— substance misuse, death distress, self-control, and bedtime procrastination—mapped against nine observed variables. The fit of the measurement model to the data was evaluated using several indices, with the following outcomes: a chi-square value of χ^2^(21, *N* = 479) = 54.58, indicating a ratio of χ^2^/df = 2.59, with a significance level of *p* <.001. The GFI stood at 0.974, the NFI at 0.963, the CFI at 0.977, and the TLI at 0.960. Additionally, the SRMR was 0.038, and the RMSEA was 0.58. These metrics collectively signify that the measurement model was satisfactorily congruent with the observed data. Moreover, the significant loadings of the indicators on their designated latent variables, varying from 0.34 to 0.90 (*p* <.001), affirm that the observed variables were appropriate and effective representations of the latent constructs under examination.

**Fig. 1:**
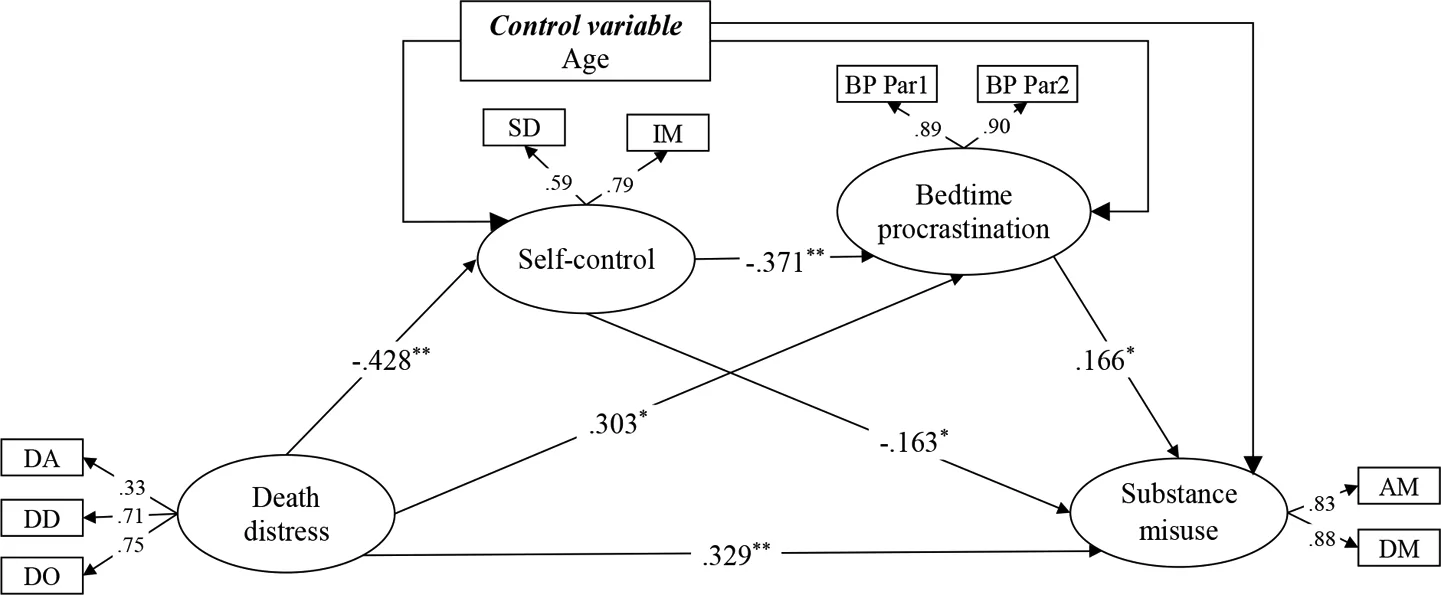
The serial mediating roles of self-control and bedtime procrastination. Note. ^*^p <.05, ^**^p <.01; DA death anxiety; DD death depression; DO death obsession; SD self-discipline; IM impulsivity; BP Par parcels of bedtime procrastination; AM alcohol misuse; DM drug misuse

Following the evaluation of measurement model, the serial mediation model was analyzed using structural equation modeling. This analysis, depicted in Fig. 1, supported satisfactory model fit indices: χ^2^(27) = 89.09, *p* <.001; χ^2^/df = 3.29; GFI = 0.958; NFI = 0.942; CFI = 0.958; TLI = 0.931; SRMR = 0.061; RMSEA = 0.069 (90% CI = 0.054–0.086). Within this model, death distress emerged as a significant predictor of substance misuse (*β* = 0.329, *p* <.001), self-control (*β* = − 0.428, *p* <.001), and bedtime procrastination (*β* = 0.303, *p* <.01). Moreover, self-control also significantly predicted substance misuse (*β* = − 0.163, *p* <.05) and bedtime procrastination (β = − 0.371, *p* <.01). As hypothesized, bedtime procrastination was found to significantly predict substance misuse (β = 0.166, *p* <.05), illustrating the complex interplay and predictive relationships between death distress, self-control, bedtime procrastination, and substance misuse within the model.

Bootstrapped serial mediation analyses were employed to assess the proposed indirect influence of death distress on substance misuse via the mediating roles of death distress and bedtime procrastination. The findings indicated that the mediating effects of both self-control (bootstrap coefficient = 0.184, with a 95% confidence interval [CI] between 0.002 and 0.452) and bedtime procrastination (bootstrap coefficient = 0.132, with a 95% CI between 0.018 and 0.323) were statistically significant. These results suggest that each mediator independently contributes to the pathway from death distress to substance misuse. Additionally, the specified mediating pathway implicating death distress leading to self-control, which in turn leads to bedtime procrastination, and subsequently to substance misuse, was found to be statistically significant (bootstrap coefficient = 0.069, with a 95% CI between 0.010 and 0.205). This supports the hypothesized serial mediation model, as detailed in Table 3.

**Table 3:** Indirect effect of death distress on substance misuse via self-control and bedtime procrastination

Path	Coefficient	95%CI	
		LL	UL
Death distress → Self-control → Substance misuse	0.184	0.002	0.452
Death distress → Bedtime procrastination →Substance misuse	0.132	0.018	0.323
Death distress → Self-control → Bedtime procrastination → Substance misuse	0.069	0.010	0.205

Note. *CI* confidence interval; *LL* lower limit; *UL* upper limit

## Discussion

Understanding the complex relationships among death distress, self-control, bedtime procrastination, and substance misuse is essential for advancing our knowledge of the psychological factors contributing to maladaptive behaviors. Previous literature has consistently highlighted the significance of each study variable in isolation, emphasizing the need to explore their interconnected relationships. In this context, the aim of the current study was to employ a serial mediation model to examine how death distress, self-control, and bedtime procrastination collectively influence the likelihood of engaging in substance misuse.

The first key finding of our study was the negative association between death distress and self-control. Consistent with previous research (Sadowski et al. [16]; Tomkins [21]), our results emphasize that individuals experiencing elevated levels of death distress may struggle with maintaining adequate self-control. Similarly, Gailliot et al. [3] observed that people with higher self-control showed fewer death-related thoughts after encountering cues related to death. Additionally, the same study showed that people with higher self-control experienced lower death distress and were less likely to perceive ambiguous situations as related to death and showed a reduced propensity for worldview defense when directly reminded of their mortality. The fear of mortality may undermine one’s ability to regulate impulses effectively, leading to a diminished capacity to resist immediate gratification or impulsive behaviors. This decline in self-control, in turn, was identified as a significant mediator in the relationship between death distress and bedtime procrastination. Our results align with the findings of Steel [8, 19], highlighting the role of self-control deficits in the procrastination process. As individuals with heightened death distress struggle to exercise self-discipline, they may find it challenging to adhere to bedtime routines, ultimately lead to bedtime procrastination.

Bedtime procrastination emerged as the second mediator in the serial mediation model, linking self-control deficits to substance misuse. Our results align with the studies of Kroese et al. [5, 7], indicating that bedtime procrastination may disrupt healthy sleep patterns and contribute to unhealthy behaviors such as substance misuse. The inability to adhere to a consistent bedtime routine may disrupt the circadian rhythm, exacerbating stress and anxiety, and increasing the likelihood of engaging in substance misuse as a maladaptive coping mechanism. As a result of their inability to get enough sleep, many turn to unhealthy coping strategies like substance abuse in order to ease their increased emotional discomfort.

Furthermore, our findings support our last hypothesis that heightened death distress indirectly contributes to substance misuse through the serial mediation of self-control and bedtime procrastination. Individuals facing existential concerns may experience a gradual impact, where compromised self-control influences bedtime procrastination, subsequently enhancing vulnerability to substance misuse. In other words, heightened death distress depletes self-control resources, leading to compromised self-control. This compromised self-control, in turn, mediates the relationship between death distress and bedtime procrastination. Bedtime procrastination, influenced by weakened self-regulation, subsequently enhances vulnerability to substance misuse through disrupted sleep patterns and increased stress and anxiety. This comprehensive understanding provides insights into the psychological mechanisms linking death distress to substance-related behaviors, offering a foundation for targeted interventions and preventive measures.

While our study provides valuable insights, it is essential to acknowledge its limitations. The cross-sectional nature of the data limits our ability to establish causality definitively. Future longitudinal studies could explore the temporal dynamics of these relationships, providing a more robust foundation for understanding the developmental pathways linking death distress, self-control, bedtime procrastination, and substance misuse. Second, the model may not account for all potential mediators in the relationships studied. There could be other variables that influence or interact with the proposed variables, and these should be considered in future research. Third, our study relies on self-report measures for variables like death distress, self-control, bedtime procrastination, and substance misuse, which may introduce common method bias and potential inaccuracies due to social desirability or recall biases. Finally, the investigation utilized a convenience sample, diminishing the generalizability of its findings. Future research should aim to use larger and more diverse samples.

In conclusion, this research contributes to the growing body of literature exploring the relationships between death distress, self-control, bedtime procrastination, and substance misuse. The findings underscore the importance of addressing existential concerns and promoting self-control as potential avenues for intervention in preventing substance misuse. Understanding the interconnected nature of these variables can guide the development of focused therapeutic strategies and public health interventions designed to alleviate the negative outcomes linked to heightened death distress.

## Data Availability

Data will be available on request.

## Declarations

### Pre-Registration Statement

This study was not pre-registered.
